# High-Performance Liquid Chromatographic Determination of Rivastigmine in Human Plasma for Application in Pharmacokinetic Studies

**Published:** 2010

**Authors:** Hossein Amini, Abolhassan Ahmadiani

**Affiliations:** a *Department of Pharmacology, Faculty of Medicine, Golestan University of Medical Gorgan, Iran.*; b *Department of Pharmacology, Neuroscience Research Center, Saheed Beheshti University of Medical Sciences and Health Services,Tehran,Iran. *

**Keywords:** Rivastigmine, Liquid- liquis extraction, HPLC, Spectrophotometric detection, Pharmacokinetics

## Abstract

A simple and reproducible HPLC method with spectrophotometric detection was developed for determination of rivastigmine in human plasma. Liquid-liquid extraction of rivastigmine and donepezil (as internal standard) from plasma samples was performed with 1-butanol/n-hexane (2:98 v/v) in alkaline condition followed by back-extraction into diluted acetic acid. Chromatography was carried out using a Silica column (250 mm × 4.6 mm, 5 μm) under isocratic elution with acetonitrile-50 mM aqueous sodium dihydrogen phosphate (17: 83 v/v, pH 3.1. Analyses were run at a flow-rate of 1.3 mL/min at of 50°C. The recovery was 90.8% and 95.7% for rivastigmine and the internal standard donepezil, respectively. The precision of the method was 2.6% to 9.1% over the concentration range of 0.5-16 ng/mL for rivastigmine in plasma with a linearity greater than 0.999. The method was specific and sensitive, with a quantification limit of 0.5 ng/mL and a detection limit of 0.2 ng/mL in plasma. The method was used for a bioequivalence study in healthy subjects.

## Introduction

Rivastigmine hydrogen tartrate, i.e. (−)*S*-*N*-ethyl-3-[(1-dimethyl-amino)ethyl]-*N*-methylphenyl- carbamate hydrogen tartrate, is a carbamate inhibitor of acetylcholinesterase (AChE) used in the treatment of mild to moderate Alzheimer’s disease in adults. It is classified as an intermediate-acting or pseudo-irreversible agent, due to its long inhibition on AChE of up to 10 h (1). The drug is rapidly (0.8-1.2 h) absorbed orally. Its elimination half-life is 1-2 h, and it is converted to an inactive metabolite at the site of action, by passing hepatic metabolic pathways (2). The oral bioavailability of rivastigmine increases from approximately 35% at 3 mg to 71.7% at 6 mg (3). 

Few analytical methods have been reported in the literature for monitoring plasma levels of rivastigmine with a limit of quantification (LOQ) of 0.2 ng/mL, which include GC-MS (3-6) and LC-MS (7-10). However, mass spectrometer is expensive and not readily available in most clinical research laboratories. In addition, some methods (3-8) require intensive work for sample preparation. Recently, an HPLC method with fluorimetric detection was also reported (11); however, its LOQ of 25 ng/mL is not satisfactory. 

Rivastigmine has UV absorption at very short wavelengths (12). This very non-specific UV absorption makes it difficult to develop a specific, selective and sensitive UV method, particularly for complex media such as plasma. In the present work, by using a new sample clean-up procedure and chromatographic separation method, a selective HPLC–UV assay method for rivastigmine in plasma was developed. The present method was found reliable and applied for a bioavailability study of rivastigmine capsules in healthy volunteers.

## Experimental

*Reagents*

Rivastigmine hydrogen tartrate and donepezil hydrochloride were obtained from Vasudha Pharma Chemicals Ltd. (Hyderabad, India). HPLC grade methanol and acetonitrile, and analytical grade n-hexane and 1-butanol were from Merck (Darmstadt, Germany). All other reagents used were also of analytical grade, obtained from Merck (Darmstadt, Germany).

*Instrumentation*

The analyses were performed by a Knauer chromatographic system (Berlin, Germany) equipped with a Smartline 1000 solvent delivery pump, Smartline 2500 ultraviolet detector (operated at 200 nm), Jet stream column heater and ChromGate integrator. The samples were injected by a Rheodyne 7725i loop injector with an effective volume of 100 μL. A Waters Spherisorb S5 W (250 mm×4.6 i.d.; 5 μm particle size) with a Waters Spheisorb S5W guard column (30 mm×4.6 mm i.d.) was used for the chromatographic separation. The mobile phase comprised of acetonitrile- 50 mM sodium dihydrogen phosphate (17: 83 v/v), adjusted to pH 3.1 with concentrated phosphoric acid and 4 M sodium hydroxide. Analyses were run at a flow rate of 1.3 mL/min at 50° C. 

*Standard solutions*

The internal standard donepezil hydrochloride was dissolved in methanol to make concentrations of 0.1 mg/mL. Stock solution of rivastigmine was prepared in methanol at a free base concentration of 5 mg /50 mL by dissolving 8 mg rivastigmine hydrogen tartrate in 50 mL of methanol. The stock solutions of the internal standard and rivastigmine were stored at -20 ºC. Working standard solutions were prepared daily from stock solutions, by dilution with 0.1% acetic acid. 

*Calibration curve and quantitation*

Seven-point standard calibration curves were obtained by dissolving appropriate amounts of rivastigmine in plasma samples. The plasma standards ranged from 0.5 to 16 ng/mL. Calibration curves were constructed by plotting peak height ratio (*y*) of rivastigmine to the internal standard versus the rivastigmine concentration (*x*). A linear regression was used for quantitation. The prepared calibration standards (1 mL) were pipetted into 4.5 mL polypropylene tubes with cap (10 × 70 mm) and stored at -20°C pending analysis.

*Extraction procedure*

Extraction was performed by adding 20 μL of the internal standard (40 ng of donepezil), 20 μL of 1 M NaOH and 3 mL of 1-butanol/n-hexane (2:98, v/v) to 1 mL of plasma in 4.5 mL polypropylene tube and shaking for 2 min. After centrifugation at 6000 g for 2 min, the whole organic layer was separated and transferred into another 4.5 mL tube. Then, 100 μL of 0.1% acetic acid was added. The mixture was vortex-mixed for 2 min and centrifuged at 6000 g for 2 min. The upper organic phase was discarded completely and a volume of 80 μL of aqueous phase was injected into the chromatographic system.

*Assay validation*

Blank human plasma obtained from 24 healthy volunteers, was assessed as described above and compared with the standard samples containing rivastigmine and also the plasma samples from volunteers after administration of rivastigmine, in order to evaluate the selectivity of the method. The precision and accuracy of the method were examined by adding known amounts of rivastigmine to pool plasma (quality control samples). Quality control samples were made from a stock solution other than that used to prepare the standards, and were not used for constructing calibration curves. For intra-day precision and accuracy, five replicate quality control samples at each concentration were assayed on the same day. The inter-day precision and accuracy were evaluated by the same results from five different days within the two-weeks period of analyzing the plasma samples of volunteers. The absolute recoveries (n = 5) were calculated by comparing the peak heights obtained from the prepared sample extracts with those found by direct injection of the same concentrations of drug in 0.1% acetic acid. The LOQ was estimated by analyzing rivastigmine at low concentrations of the calibration curve. The LOQ was defined as the minimum concentration where accuracy and precision were still better than 10%. To determine the limit of detection (LOD), plasma concentrations lower than the minimum end of the calibration curve were used. The LOD was then defined as the minimum concentration which caused a signal three times the noise (S/N=3/1). 

The stability of rivastigmine was assessed for standard and volunteers’ samples stored at -20°C for up to 3 months. The acceptance margins for all stability tests were ±10% of the original concentration. 

*In-vivo pharmacokinetic study*

The assay was used for a comparative bioavailability study of two capsule preparations containing 3 mg rivastigmine. The study protocol was approved by the Ethics Committee of the Ministry of Health of Iran, and written informed consent was signed by volunteers. 

Twenty four healthy volunteers participated in the study. The study was conducted using a two-way cross over design, and as a single dose randomized trial. The two formulations were individually administrated on two different days separated by a washout period of 7 days, between the two study medications, to fasted subjects. Food and drinks were not allowed until 3 h after ingestion of the capsules. Multiple blood samples (5 mL) were collected before and 0.25, 0.5, 0.75, 1, 1.5, 2, 2.5, 3, 3.5, 4, 5, 6 and 8 h after the dosing. The plasma was immediately separated by centrifugation and frozen at −20 ◦C until analysis. 

A non-compartmental analysis was used in data processing. Maximum plasma concentration (C_max_) and time to C_max_ (t_max_) were determined by inspection of the plasma concentration-time curves. Elimination constant (k_el_) was determined by means of least-squares regression of the data from the last 4-6 points of each plasma concentration-time curve. Plasma half-life (t1/2) was calculated as ln(2)/k_el_. AUC_0-t_, the area under the concentration-time curve from time zero to the last detectable drug concentration (C_t_), was calculated by the linear trapezoidal rule. The AUC from time zero to infinity (AUC_∞_) was calculated by adding AUC_0-t_ to the extrapolated AUC, obtained by dividing C_t _by k_el_. 

## Results and Discussion

*Method development*

*UV detection*

Significant UV absorption for rivastigmine was obtained at wavelengths below 220 nm. Spectrophotometric detection was used at 200 nm, since at this wavelength rivastigmine gave 1.5, 3 and 6 times higher peaks in comparison to 210, 215 and 220 nm, respectively. 

*Selection of chromatographic column*

Rivastigmine is usually analyzed on a C18 column (7- 9, 11-12). In the present study, retention of rivastigmine and some other drugs was examined on C18, CN and silica columns with mobile phases consisting of mixtures of phosphate buffer and acetonitrile. Interestingly, the results showed that although rivastigmine and zolpidem (as the other tested drugs) had strong retentions on C18 column, they had slightly more retentions on silica column in comparison to CN column (Table 1).

**Table1:** The retention of rivastigmine, zolpidem, donepezil and norverapamil on different chromatographic columns

**Drug **	***K ′ on column ***		
	C18	CN	*Silica *
Rivastigmine	3.2	0.8	0.9
Zolpidem	6.4	1.6	1.9
Donepezil	ND*	3.3	1.1
Citalopram	ND*	3.3	0.4
*Norverapamil *	ND*	8.4	0.5

ND= not detected

 These results show that for rivastigmine and zolpidem, in addition to reversed-phase retention, other retention mechanisms such as hydrogen-binding or ion- exchange are probably involved. While other drugs and also endogenous plasma interferences had extremely lower retentions in Silica column in comparison to C18 or CN column, unusual retention of rivastigmine in Silica column offered selectivity and more importantly, excellent clear chromatograms. Therefore, a silica column was selected for rivastigmine assay in plasma. 

Neither the efficiency of the Silica column nor the retention of rivastigmine and the internal standard changed significantly after more than 3000 biological sample injections. However, the guard column should be replaced after every 200 injections. 

*Mobile phase*

A mobile phase containing acetonitrile was the first choice at 200 nm, since it has a lower UV cut-off than methanol. The separation of rivastigmine by varying mobile phase compositions was investigated. A simple buffered acetonitrile mobile phase with an acidic pH was found appropriate for the separation. Addition of triethylamine (a silanol blocking agent) to the mobile phase reduced sharpness of rivastigmine peak and therefore was avoided as a mobile phase component. 

*Temperature*

Higher temperatures increased the column efficiency for rivastigmine and therefore were used in the present study. Since high temperature may reduce column life-time, temperatures above 50°C were not tried. 

*Sample preparation*

Rivastigmine could be extracted from plasma, using methyl tert-butyl ether (MTBE) (3, 9). Based on our experiences; MTBE simultaneously extracted a lot of plasma interferences and was not suitable for HPLC-UV assay of rivastigmine. For the same reason, ethyl acetate and dichloromethane were also not suitable. A highly selective extraction of rivastigmine was obtained, using 1-butanol/n-hexane (2:98 v/v). Higher percentage of 1-butanol in n-hexane was not suitable, since recovery of rivastigmine was reduced in back-extraction and also plasma interferences appeared in chromatograms. Evaporation of the extraction solvents produced interferences in chromatogram and therefore, a back-extraction was used. While back-extraction into phosphoric acid or perchloric acid produced incomplete recovery, back-extraction into diluted acetic acid gave reproducible and high recovery of rivastigmine and the internal standard donepezil. Diluted acetic acid as back-extraction medium also produced more clear chromatograms than did phosphoric acid and perchloric acid. 

*Selection of internal standard*

Among drugs tested as internal standard, zolpidem had a similar retention behavior to rivastigmine in different chromatographic columns. However, its recoveries was not suitable using the selected extraction procedure. In contrast, donepezil, citalopram and norverapamil had high recovery in extraction from plasma using the proposed method. Donepezil was selected as the internal standard due to its higher retention in silica column, in comparison with citalopram and norverapamil. 

*Assay validation*

**Figure 1:**
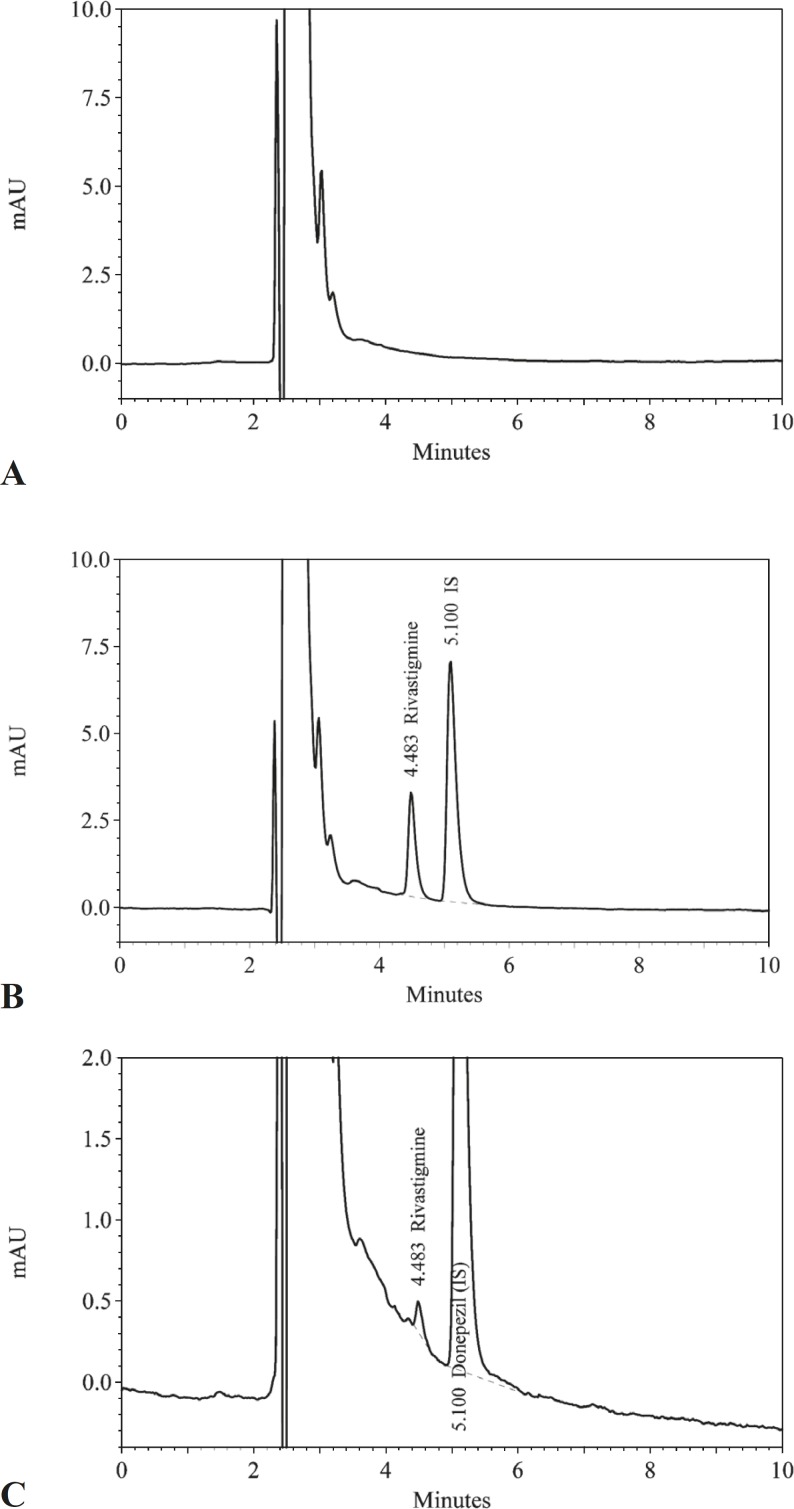
Representative chromatograms of (A) a blank plasma; (B) plasma containing 12 ng/mL rivastigmine; (C) a volunteer plasma sample, 5 h after taking 3 mg capsules of rivastigmine (0.823 ng/mL).

Representative chromatograms of drug-free plasma, plasma containing dissolved rivastigmine, and plasma samples from volunteers collected after oral dosing with rivastigmine are shown in Figure 1. 

The retention times for rivastigmine and the internal standard were 4.5 and 5.1 min respectively. No interfering peaks from the endogenous plasma components were observed at the retention time of rivastigmine or internal standard. In addition, no late-eluting peak was observed and new samples could be injected every 6 min. Several drugs including azithromycin, omeprazole, ranitidine, cimetidine ciprofloxacin, ofloxacin, amoxicillin, cefixime, clavulanic acid, valproic acid, metformin, acyclovir, diazepam, oxazepam, moclobemide, prazosin, terazosin, loratadine, cyclosporine, zolpidem, citalopram, sumatriptan, rizatriptan, verapamil and clonazepam were tested and none of them interfered. The calibration curves were linear over the concentration range of 0.5–16 ng/mL in human plasma, with a correlation coefficient greater than 0.999. The limit of quantification was 0.5 ng/mL and the limit of detection was 0.2 ng/mL. The results of the intra- and inter-day accuracy and precision determination have been presented in Table 2. 

**Table 2:** The accuracy, within- and between-day precision and recovery data for the measurement of rivastigmine in human plasma (n=5)**.**

**Nominal **	**Recovery **	**Intra-day **			**Inter-day **		
Concentration (ng/mL)	(%)	Mean±SD	Precision (%)	Accuracy (%)	Mean±SD	Precision (%)	*Accuracy (%) *
0.5	89.7 ± 6.6	0.512 ± 0.031	6.1	2.4	0.47 ± 0.04	9.1	-5.6
2.0	93.6 ± 3.3	1.86 ± 0.12	6.4	-7.0	1.93 ± 0.11	5.7	-3.5
8.0	86.3 ± 2.3	8.09 ± 0.24	3.0	1.1	8.14 ± 0.31	3.8	1.8
16.0	93.5 ± 1.3	15.69 ± 0.41	2.6	-1.9	16.46 ± 0.53	3.2	2.8

ND = not determined (K ′ was longer than 12)

Mobile phase = Acetonitrile-0.05 M phosphate (17: 83 v/v, pH=3.1)

The RSDs of intra-day precision ranged between 2.6 and 6.4%, whereas that of inter-day precision were between 3.2 and 9.1%. The intra-day mean error between -7.0 and 2.4%, whereas the inter-day mean error was between -5.6 and 2.8%. The mean absolute recoveries for rivastigmine and internal standard using the present extraction procedure were 90.8 and 95.7%, respectively. Stability properties of rivastigmine during storage and freeze-thaw cycles have been reported (7-10). In the present study, the stability studies demonstrated stability of rivastigmine in human plasma samples for at least 3 months storage at -20°C. 

*Pharmacokinetic results*

**Figure 2:**
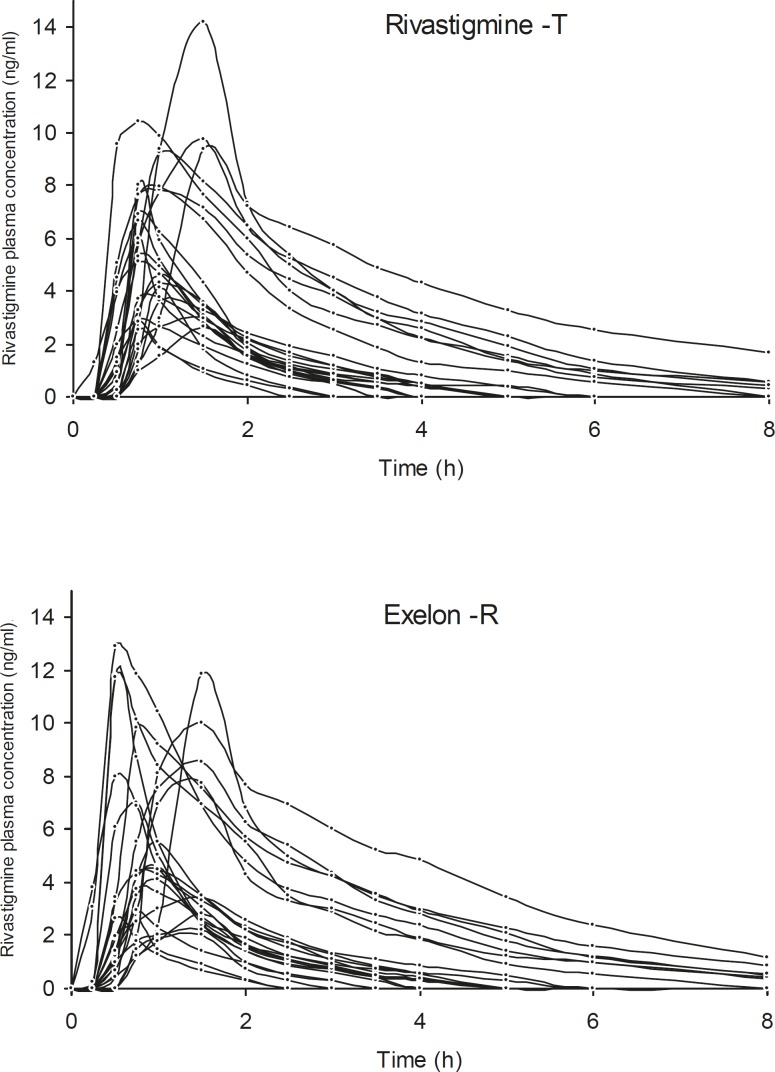
Plasma concentration-time profiles of rivastigmine in 24 healthy volunteers following oral administration of 3 mg capsule of Exelon and a test product in a cross over study

The proposed method was used for the determination of rivastigmine in plasma samples bioequivalence study. The plasma rivastigmine profiles for volunteers after taking two products are shown in Figure 2. The pharmacokinetic parameters obtained from the two preparations have been summarized in Table 3. The extrapolated fraction of the AUC_0-∞_ accounted for only 8-9%, which indicates a suitability of the analytical method for pharmacokinetic studies.

**Table 3:** Phamacokinetic parameters (as mean ± SD) for rivastigmine, following oral administration of the test or reference (Exelon) capsules at a dose of 3 mg, in 24 healthy adult male volunteers

**Group**	**C**_max_ ( ng/mL)	**t**_max_ (h)	**AUC**_0-t_ ( ng/mL•h)	**AUC**_0-∞_ (ng/mL•h)	**t**_1/2_ (h)
Test product	6.02 (±2.94)	0.99 (±0.29)	11.78 (±8.98)	12.71 (±9.89)	1.09 (±0.52)
Exelon-R	6.27 (±3.53)	0.98 (±0.41)	11.95 (±9.30)	12.79 (±9.96)	1.11 (±0.51)

C_max_ = maximum plasma concentration; t_max_ = time to reach C_max_; AUC_0-∞ _= area under the concentration-time curve (AUC) from time zero to infinity; AUC_0-t _= AUC from time zero to the last measurable concentration; t_1/2 _= elimination plasma half-life

In conclusion, it could be said that a selective and sensitive HPLC-UV method for quantification of rivastigmine in human plasma has been developed and validated. The simple extraction procedure is based on liquid–liquid extraction followed by back-extraction into diluted acid. The method is time-saving and cost-effective, and provides the best alternative for mass spectrometry which is quite expensive and still not simply available in most laboratories. The sensitivity of the assay is sufficient to follow the pharmacokinetics of rivastigmine after administration of a low dose of rivastigmine to human subjects.
